# Caffeine affects autonomic control of heart rate and blood pressure recovery after aerobic exercise in young adults: a crossover study

**DOI:** 10.1038/s41598-017-14540-4

**Published:** 2017-10-26

**Authors:** Luana Almeida Gonzaga, Luiz Carlos Marques Vanderlei, Rayana Loch Gomes, Vitor Engrácia Valenti

**Affiliations:** UNESP - Univ Estadual Paulista, Postgraduate Program in Physiotherapy, Presidente Prudente – São Paulo, 19060-900 Brazil

## Abstract

The post-exercise recovery period is associated with changes in autonomic modulation, which can promote an intercurrent-favorable environment. Caffeine has the ability to release catecholamines, but its effects after exercises is little explored. The present study aims to evaluate the acute effects of caffeine on the autonomic control and cardiorespiratory parameters after moderate intensity aerobic exercise. 32 young males (23,59 ± 3,45 years) were submitted to two protocols: Placebo and Caffeine, consisting of 15 minutes of rest, 30 minutes of exercise on a treadmill to 60% on VO2peak, followed by 60 minutes of recovery. Heart rate variability indices and cardiorespiratory parameters were determined at different times during the protocols. The RMSSD and SD1 indices recovered faster in placebo (p < 0.05). The systolic blood pressure differences were found from the 1st to the 5th minute of recovery with the caffeine protocol and from the 1st and 3rd minute with the placebo, whereas, for diastolic blood pressure, significant differences (p < 0.0001) were observed only for the caffeine protocol at the 1st and 3rd minutes of recovery. Caffeine was shown to be capable of delaying parasympathetic recovery but did not influence the behavior of the respiratory rate, oxygen saturation or frequency-domain HRV indices.

## Introduction

Caffeine is one of the most consumed substances in the world. It is present in various foods, such as coffee, teas, energy drinks, supplements and medicines^1^ and is known for its psychoactive and ergogenic capacity in long-term exercise^2^. The physiological effects of caffeine have been widely studied^3^ and can be attributed to its ability to stimulate the nervous system. Due to its structural similarity, caffeine can lead to the blockading of adenosine receptors (A1 and A2)^4^ and increase autonomic nervous system (ANS) activity through the release of plasma catecholamines, inducing tachycardia and increased blood pressure (BP)^3,5,6^.

The effects of caffeine during physical exercise have been explored^3^ because of its stimulatory action, with fatigue stalling and improved performance^7,8^. However, few studies have evaluated its effects during post-exercise recovery^7–10^, which is a critical period in which several modifications occur, among them changes in autonomic modulation^11^, which may promote an environment conducive to the development of rhythm disturbances, abnormal responses of heart rate (HR) and BP in predisposed individuals^11,12^.

Nevertheless, most of the studies that have evaluated the effects of caffeine supplementation on post-exercise recovery were performed with protocols of progressive and exhaustive exercises^7–9^, studying changes in physiological variables such as HR, BP, oxygen consumption and autonomic modulation^7–10^.

However, results are still inconclusive. Some studies reported lower vagal reactivation and increased sympathetic activity during the recovery period^7,9^, while others did not observe expressive differences^8,10^. The same is true for cardiorespiratory variables, with changes in BP and HR during the recovery period^7^, as well as the absence of alterations^8^.

Taking moderate intensity aerobic exercise as the type of exercise modality closest to the normal practice of physical activity most recommended for the promotion of health^13^, we hypothesize that caffeine promotes a slower recovery of cardiorespiratory parameters and ANS, assessed by means of HRV linear indices. We aim to evaluate the influence of caffeine on the recovery of autonomic heart rate control, through linear HRV indexes and cardiorespiratory parameters after moderate intensity aerobic exercise.

## Methods

### Participants

Forty healthy young male volunteers (23.59 ± 3.45 years), were recruited through social network ads. Smokers, alcoholics, individuals with known cardiovascular, respiratory or neurological disorders or with musculoskeletal injury that would impede the accomplishment of the exercise protocols were not included. Individuals who participate in an organized team or individual sport requiring regular competition were not included. Volunteers who showed a series of RR intervals with less than 95% of sinus beats and those who did not complete all stages of the experimental protocol were excluded.

All volunteers signed a consent letter and were informed of the procedures and objectives of the study. The study’s procedures were all approved by the Research Ethics Committee of the Faculty of Philosophy and Sciences at the Paulista State University, Marilia – São Paulo, SP, Brazil (file no. CEP-2200/11) and conformed with resolution 466/12 of the National Health Council of 12/12/2012. The present study’s crossover clinical trial is registered in the Clinical Trials network by the identification code NCT02917889 on September 19th, 2016.

### Study Design

The protocols were performed between 5:30 and 9:30 PM to standardize the circadian influence and with temperature between 23 °C and 24 °C and humidity between 60% and 70%. Prior to the experimental procedure, all volunteers were advised to abstained from caffeinated and alcoholic beverages, food, and strenuous exercise for at least 24 hours before each testing session and consume a light meal two hours before the experiment.

The experimental procedure was divided into three stages with a minimum interval of 48 hours between them, in order to allow adequate recovery time for the participants. Before the beginning of the first stage, body weight measurements on a digital scale (Welmy W 200/5, Brazil) and height in stadiometer (ES 2020 - Sanny, Brazil) were recorded.

The first stage of the experimental procedure consisted of the maximum effort test. This was performed first as it was used to determine the exercise intensity in subsequent stages. The other steps were called the placebo protocol and caffeine protocol, whose order of execution was established through a randomization process using a coin. The volunteers were blinded during their protocol and were not informed of the order of these protocols; however the researcher was not blinded at any time in the study.

### Maximum stress test

In order to prescribe the intensity of the exercise, the cardiopulmonary exercise test in treadmill (Inbramed, MASTER CI, Brazil) was performed using the Bruce incremental protocol^14^. The analysis of expired gases was performed using the Quark PFT commercial system (Comend, Rome, Italy), obtaining the peak oxygen consumption (VO_2_peak) established as the highest oxygen consumption achieved during the test.

### Caffeine and Placebo Protocol

Before initiating these protocols, the HR receiver (Polar RS800CX, Finland) was strapped to the volunteers to register HR beat-to-beat, followed by an intake of 300 mg of caffeine (this concentration is within the maximum allowed daily by the Food and Drug Administration (FDA)^1^) or a placebo (300 mg starch) in identical capsules according to the protocol selected. After ingesting the capsules, volunteers performed an initial supine rest for 15 minutes, HR values, systolic blood pressure (SBP) diastolic blood pressure (DBP), respiratory rate (RR) and pulse oxygen saturation (SpO_2_) being registered in the 15^th^ minute.

After these measurements, the volunteers exercised on the treadmill at a speed of 5 km/h and slope of 1% in the first 5 minutes for warm up, followed by 25 minutes with work load equivalent to 60% of the VO_2_peak HR, with the same inclination. At the end of the activity, the volunteers were again placed in the supine position and were monitored over 60 minutes, their HR, SBP, DBP, RR and SpO_2_ being logged in the 1^st^, 3^rd^, 5^th^, 7^th^, 10^th^ and from then every 10^th^ minute until the end of the recovery period. A single evaluator performed these measurements throughout the experiment to avoid errors in the measurements of the parameters evaluated.

### Blood pressure

Verification of the SBP and DBP was made indirectly with the use of a stethoscope (Littman Classic II, Saint Paul, USA) and aneroid sphygmomanometer (Welch Allyn Tycos, New York, USA) on each volunteer’s^15^ left arm.

### Respiratory rate and oxygen saturation

The RR measurements were taken by counting volunteers’ breaths for one minute without the volunteers having knowledge of the process, so that no change occurred in the respiratory pattern. The measurement of SpO_2_, was obtained by pulse oximeter (PM-50 Mindray, China).

### Analysis of HRV

The HRV analysis was registered during the whole experimental protocol by an HR receiver (Polar RS800CX, Finland), the equipment previously used to record the pulse rate of HR^16^. The HRV indices were determined at the following times: 10^th^ to 15^th^ minute of rest and during recovery (Rec): Rec1 (0 to 5 minutes), Rec2 (5 to 10 minutes), Rec3 (15 to 20 minutes), Rec4 (25–30 minutes), Rec5 (35 to 40 minutes), Rec6 (45 to 50 minutes) and Rec7 (55 to 60 minutes). During the autonomic evaluation, the volunteers were instructed to remain awake, in silence, breathing normally while resting in the supine position.

The recording examined had at least 256 consecutive RR intervals and underwent a digital filtering supplemented by manual filtering, to eliminate artifacts and only series with more than 95% of sinus heart rate were included in the study. Linear methods in time and frequency domain were applied to analyze HRV. In the time domain indexes RMSSD (square root of the average of the square of the differences between normal adjacent RR intervals) and SDNN (standard deviation of the average of all normal RR intervals) were used. The indexes of the Poincaré plot: SD1 (standard deviation of the instantaneous rate variability the rhythm) and SD2 (long-term standard deviation of R-R intervals ushers)^17^ were also calculated.

The spectral components LF and HF, in ms² and the normalized unit extracted from the Fast Fourier Transform, plus the ratio between these components (LF/HF), were used to analyze HRV in the frequency domain. The frequency bands used for each component were: low frequency (LF = 0.04–0.15 Hz) and high frequency (HF = 0.15–0.40 Hz)^17^. HRV analysis software (Kubios, Biosigna Analysis and Medical Image Group, Department of Physics, University of Kuopio, Finland)^18^ was used to analyze the linear indices in the time and frequency domains and the Poincaré plot.

### Data analysis

The sample calculation was made using a pilot test utilizing software contained in the online site www.lee.dente.br, considering the RMSSD index as variable. The scale of significant difference was taken to be 12 ms with a standard deviation of 16.2 ms. The size of the sample was 28 volunteers with a 5% significance level and an 80% test power.

Gaussian distribution of the data was verified using the Shapiro-Wilks test. For the data analysis we used descriptive statistics of the characterization of the sample, results were presented with average values, and minimum and maximum standard deviation. Comparisons of the values of the indexes of the HRV and cardiorespiratory parameters between caffeine vs. placebo protocols and moments were made by two-way repeated measures ANOVA. The repeated measures data were checked for sphericity violation using Mauchly’s test and the Greenhouse-Geisser correction was conducted when sphericity was violated.

For analysis of points in time (rest vs. recovery) we applied ANOVA for repeated measurements followed by the Bonferroni post-test for parametric distribution or the Friedman test followed by Dunn’s post-test for non-parametric distribution. Statistical significance was set at 5% for all analyses.

The analyses were performed using the Minitab software-version 13.20 (Minitab, PA, USA), GraphPad Instat – version 3.01, 1998 (GraphPad Software, Inc., San Diego California USA) and IBM SPSS Statistics version 22.0 (SPSS Inc., Chicago, IL, USA).

## Results

### Participants

**Figure 1:**
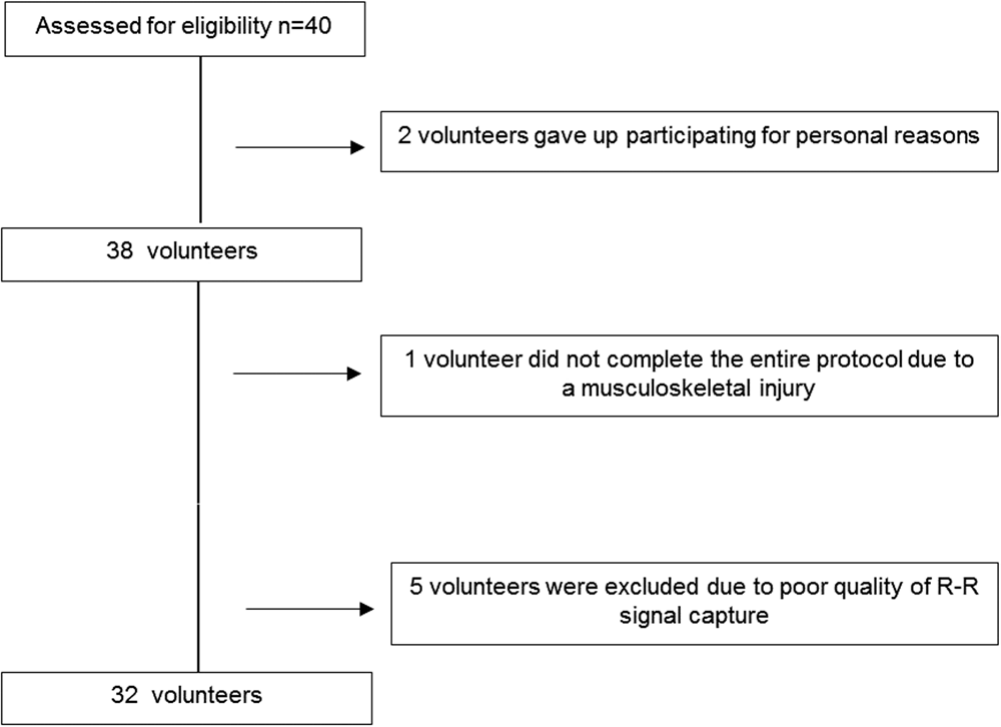
Flowchart sample loss during the study.

The anthropometric characteristics of the 32 participants and the responses obtained during the cardiopulmonary exercise testing are described in Table 1, while Fig. 1 shows the flow diagram displaying the progress of all participants through the trial. As a result of randomization, 15 volunteers started with the placebo protocol and 17 with the caffeine protocol.

**Table 1:** Mean values, followed by their respective standard deviations, minimum and maximum values of the anthropometric variables and the cardiopulmonary exercise testing.

Variables	Mean ± SD	Minimum - Maximum
Age (years)	23.59 ± 3.45	[19–30]
Height (m)	1.79 ± 7.14	[1.59–1.96]
Weight (kg)	78.87 ± 12.14	[56–100]
BMI (kg/m²)	24.40 ± 2.82	[18.28–27.70]
VO_2_peak (ml/kg/min)	44.00 ± 12.25	[23.12–72.79]
Peak HR (bpm)	185.97 ± 11.53	[154–211]
60% peak HR (bpm)	111.50 ± 13.33	[92–126]

SD = standard deviation; BMI = body mass index; kg = kilogram; m = meter; VO_2_peak = peak oxygen consumption; ml = milliliter; min = minutes; HR = heart rate; bpm = beats per minute.

### Cardiorespiratory Parameters

**Figure 2:**
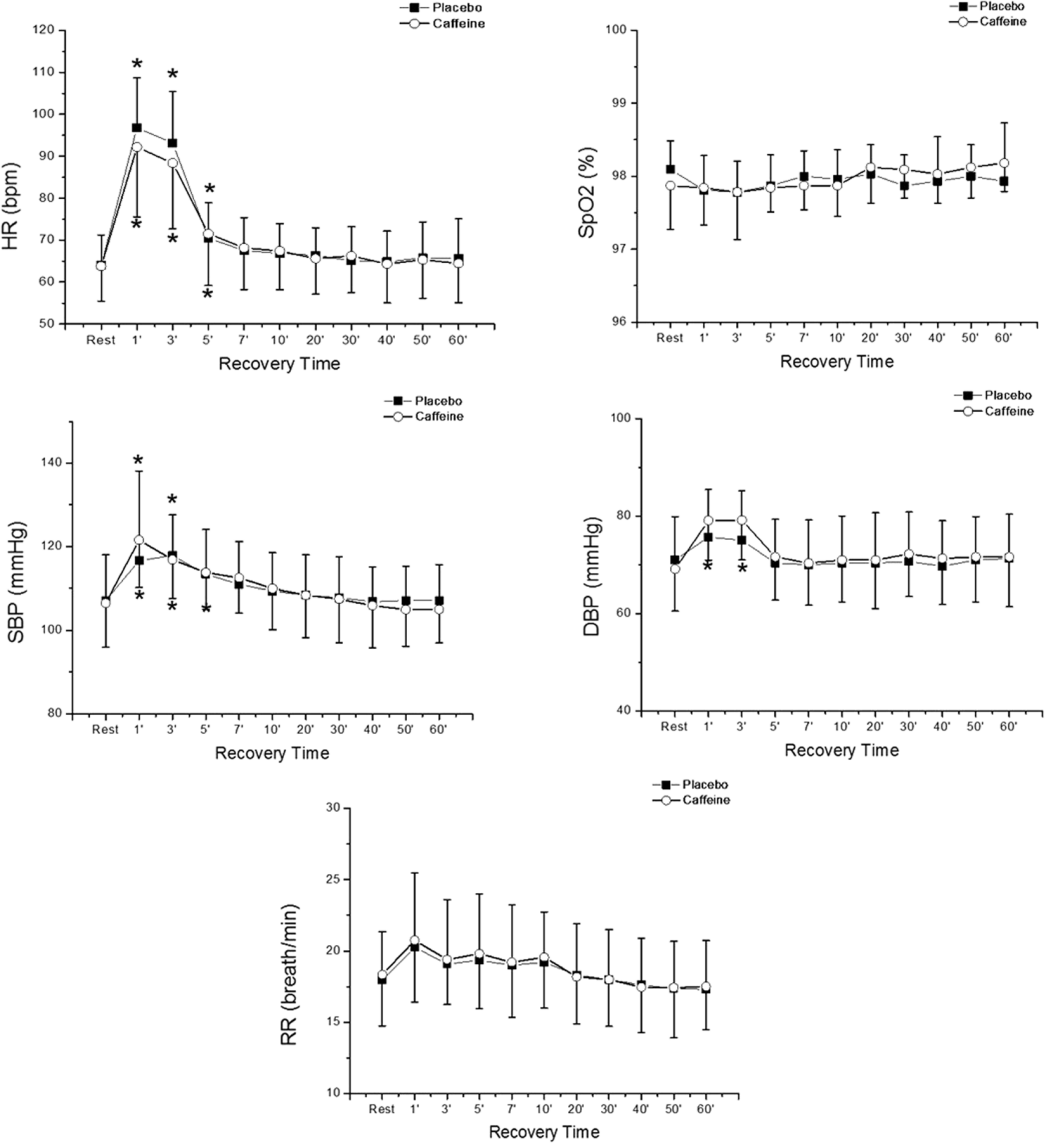
Mean values and respective standard deviations of heart rate (HR), oxygen saturation (SpO2), systolic blood pressure (SBP), diastolic blood pressure and (DBP) respiratory rate (RR) obtained in the placebo and caffeine protocols at rest and during recovery (Rec). *Values with significant difference in relation to rest (Friedman test followed by the Dunn test, p < 0.05).

The behavior of cardiorespiratory parameters during the recovery period in relation to the initial rest is presented in Fig. 2. We observed the effect of time for HR (p < 0.0001), SBP (p < 0.0001) and DBP (p < 0.0001), however, there was no effect among the protocols (HR - p = 0.666; SBP - p = 0.991; DBP - p = 0.610) and in protocol interaction (HR - p = 0.141; SBP - p = 0.313; DBP - p = 0.159) for these variables.

Significant differences were observed for HR in both protocols up to the 5^th^ minute of recovery compared to rest. Regarding the SBP responses, significant differences between recovery times and rest can be observed from the 1^st^ to the 5^th^ minute for the caffeine protocol and at the 1^st^ and 3^rd^ minutes for the placebo protocol, whereas for the DBP significant differences can be observed only for the caffeine protocol at the 1^st^ and 3^rd^ minute of recovery.

The SpO_2_ showed no effect of time (p = 0.352), protocol interaction (p = 0.287) and no effect among protocols (p = 0.279). In relation to RR, there was an effect of time (p < 0.0001), but without differences between rest and recovery, no significant differences were found for protocol interaction (p = 0.982) and no effect among protocols (p = 0.790).

### Differences between HRV indices

**Figure 3:**
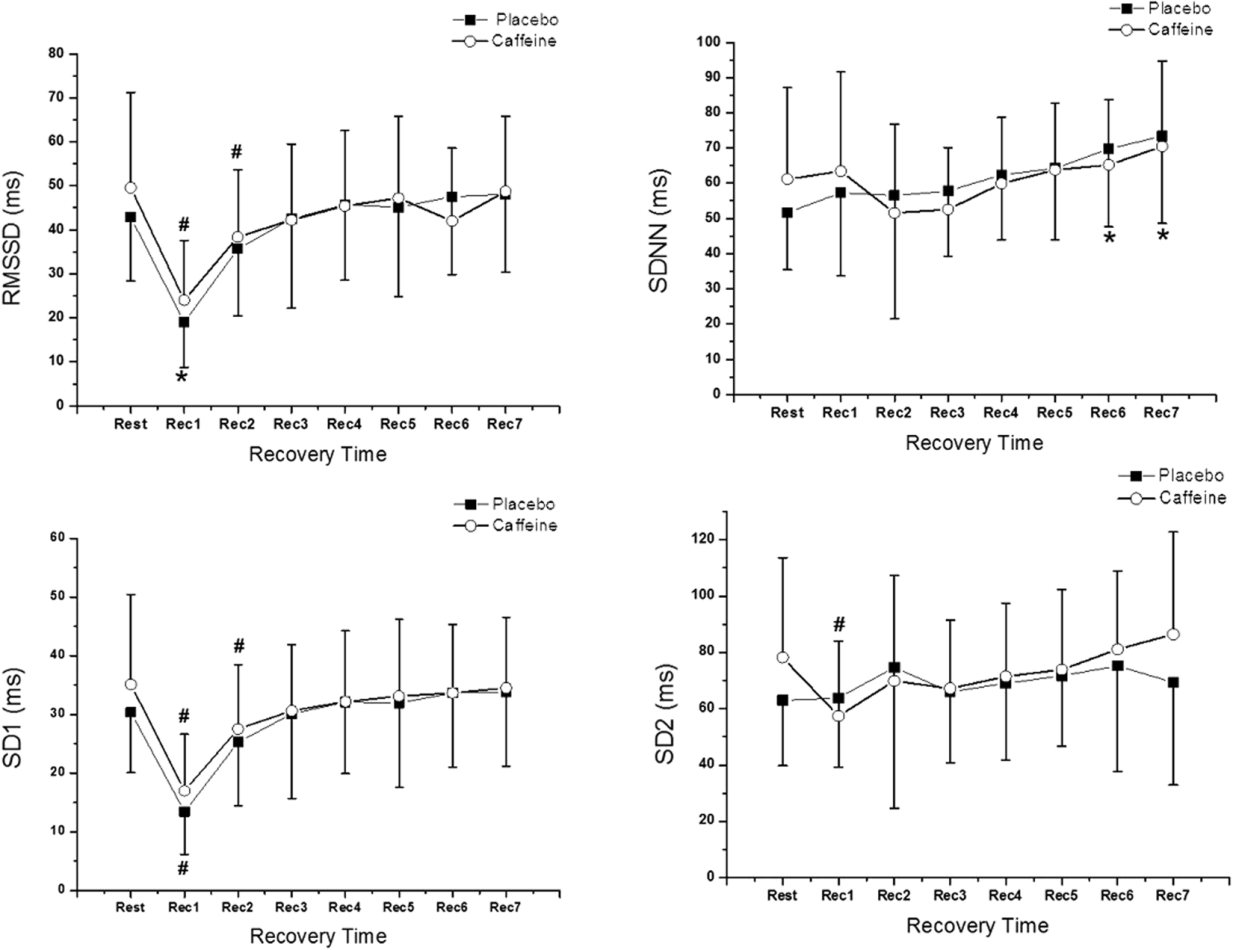
Mean values and their respective standard deviations of indices in the time domain during rest and recovery (Rec), obtained from the placebo and caffeine protocols. *Values with significant differences in relation to rest (Friedman test followed by the Dunn test; p < 0.05); ^#^Values with significant differences in relation to rest (ANOVA for repeated measures followed by the Bonferroni test, p < 0.05).

Figure 3 shows the behavior of the time domain HRV analysis during the recovery period and their comparison over the initial rest in both protocols. We observed the effect of time for the SDNN, RMSSD, SD1 and SD2 indices (p < 0.05), however no effects were observed in protocol interaction (SDNN - p = 0.209, RMSSD - p = 0.331, SD1 - p = 0.386, SD2 - p = 0.183) and no effect among the protocols (SDNN - p = 0.870, RMSSD - p = 0.559, SD1 - p = 0.533, SD2 P = 0.374). For the RMSSD and SD1 indices, significant differences were observed in Rec1 and Rec2 for the caffeine protocol but only in Rec1 for the placebo protocol. Compared to the rest, the SD2 index presented differences only in the caffeine protocol in Rec1, while the SDNN index presented differences in the placebo protocol in Rec6 and Rec7.

**Figure 4:**
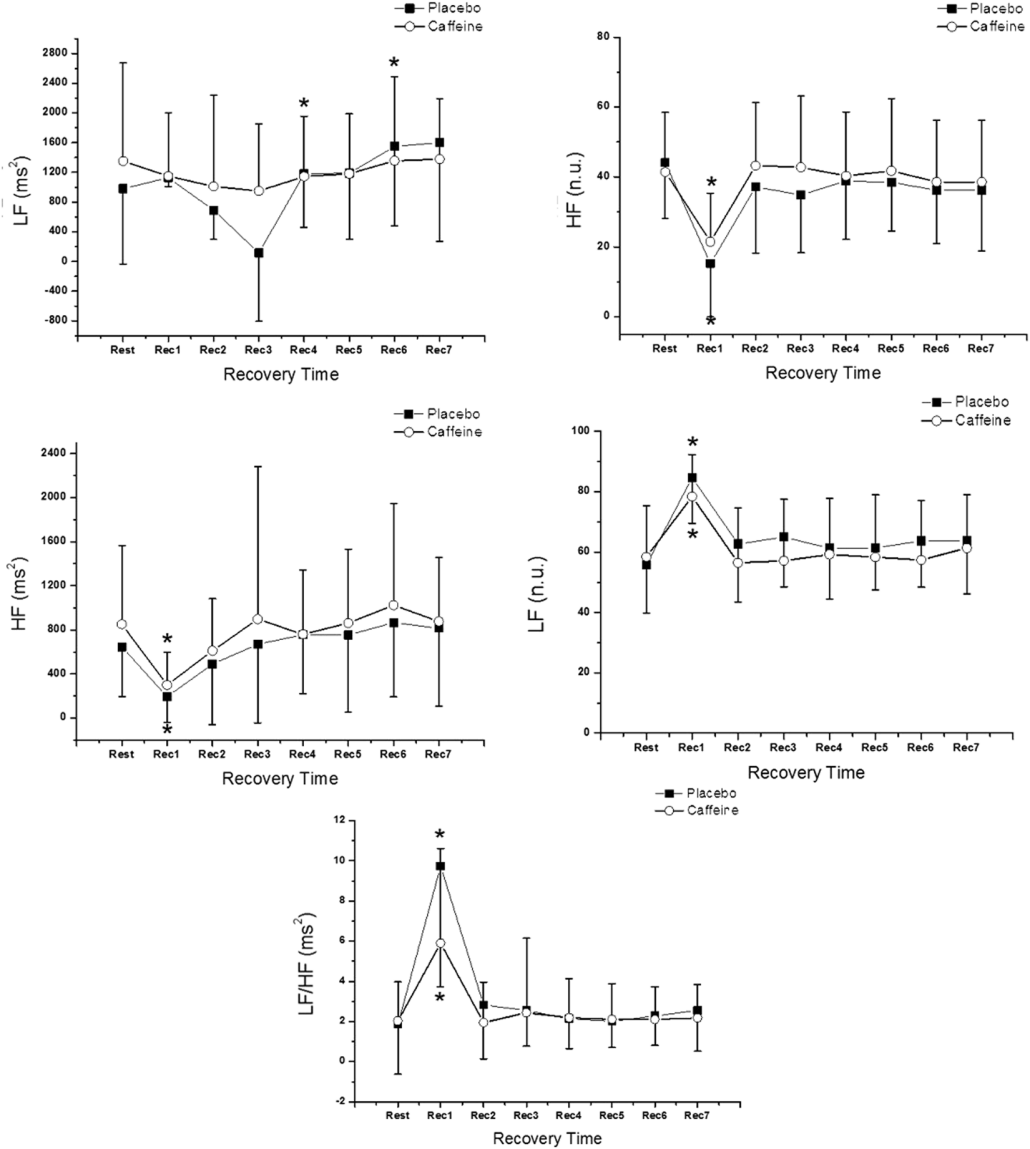
Mean values and their respective standard deviations of indices in the frequency domain in the rest period and during the recovery (Rec), obtained from the placebo and caffeine protocols. *Values of the control protocol with significant difference in relation to rest (Friedman test followed by the Dunn test, p < 0.05).

The behavior of frequency-domain HRV indices in the protocols performed can be seen in Fig. 4. The effect of time for all indices in the frequency domain can be observed (p < 0.05). No protocol interaction was observed in LF ms² (p = 0.260); HF ms² (p = 0.873); LF n.u. (p = 0.258); HF n.u. (p = 0.226). We noted protocol interaction only for the LF/HF ratio (p = 0.005). No effect among protocols was observed for the frequency domain indices (p > 0.05). For HF ms², LF n.u., HF n.u. and LF/HF indices, significant differences were observed only in Rec1 for both protocols. For LF ms2, significant differences were observed in the caffeine protocol in Rec4 and Rec6 compared to rest.

## Discussion

The present study, to our knowledge, is the first to evaluate the influence of caffeine on the recovery of autonomic heart rate control through linear HRV indexes and cardiorespiratory parameters after moderate intensity aerobic exercise. The main findings showed that caffeine: (a) was able to delay the recovery of parasympathetic heart rate control, evaluated by RMSSD and SD1 indices, (b) also promoted a later return of SBP and DBP at baseline conditions. However, caffeine did not influence HR, RR and SpO_2_.

The literature indicates that caffeine consumption increases resting values in relation to BP^6,19^. In this context, one of the mechanisms related to vasodilation in the peripheral musculature during physical exercise, resulting in post-exercise hypotension, is the release of adenosine by the active tissues. Some researchers have reported that this effect can be attenuated with caffeine use by the blockade of the receptors of adenosine A2, thus minimizing the post-exercise hypotensive effect^19,20^.

In our study, with the placebo protocol, SBP recovery was observed 5 minutes after exercise. No significant differences were found for DBP between rest and recovery. However, with the caffeine protocol, SBP and DBP showed a later recovery, only after the 7^th^ and 5^th^ minute, respectively. Results similar to ours for DBP can be found in the study by Bunsawat *et al*., who observed that DBP values remained high for the entire 30 minutes of the recovery period after maximal exercise, which was attributed to sympathetic nerve stimulation potentiated by caffeine^7^. It is worth emphasizing that the greater the intensity of exercise, the greater the stimulation of the sympathetic nervous system to meet the demands imposed by physical stress^21^.

An *et al*. found no difference in BP values during recovery after maximal exercise after the volunteers consumed a caffeine-containing energy drink at a concentration of 1.25–2.5 mg/kg^8^. It seems that the increase in BP response in exercise caused by caffeine may be related to the dose^22^, as reported by the authors, and that these alterations can also be mediated by exercise intensity^3,22^.

During exercise recovery HR decreases in a mono-exponential way^23^, and this behavior is related to the reactivation of parasympathetic activity, the level of physical condition and exercise intensity^24^, its recovery in the 1st minute after exercise being regarded as an important predictor of mortality^25^. HR recovery was observed in both protocols from the 7th minute of recovery.

Similar responses were observed by An *et al*. The authors found no effect among the protocols (control protocol vs. caffeine protocols - concentrations of 1.25 and 2.5 mg/kg)^8^. In the study by Bunsawat *et al*. there were similar HR reductions in the 1^st^ minute of recovery in both placebo and caffeine protocols, however, from the 2^nd^ minute of recovery the values were higher in the caffeine protocol, which was attributed to a greater sympathetic nervous stimulus and a greater performance in the stress test attributed to the consumption of caffeine (400 mg in capsules)^7^.

Regarding SpO_2_ and RR, no significant difference was found. All volunteers presented adequate SpO_2_ values and the physiological behavior of RR during the protocols which would be expected in healthy individuals with no related cardiopulmonary disease.

Findings in relation to autonomic heart rate control show that, although no significant difference in baseline values was found between the protocols, the use of caffeine led to a later recovery of the RMSSD and SD1 indices, occurring in Rec2 (5^th^ to 10^th^ minutes of recovery) for the placebo protocol and in Rec3 (15^th^ to 20^th^ minutes of recovery) for the caffeine protocol. Considering that these indexes predominantly reflect the parasympathetic component of heart rate regulation, these results suggest that caffeine delays the recovery of vagal heart rate control after exercise.

During physical exercise, HR and BP control are modified; the baroreflex is attenuated at the brainstem level due to the metaborreflex activation. Metabolic accumulation, as a result of elevated cellular metabolism, promotes the activation of the metaboreceptors. As a consequence, the stimulation of the non-myelinated afferent fibers leads to sympathetic activity, resulting in an increase in HR, cardiac output, BP and vasoconstriction in non-active muscles^24^.

Previous studies show that caffeine has the effect of promoting sympathetic stimulation^6,26^, which also occurs during physical exercise^27^, where a higher BP and HR response are observed when compared to the control group^7^. The delayed recovery of parasympathetic heart rate control shortly after exercise, as seen by the responses of the RMSSD and SD1 indices, can be attributed to a sympathetic-vagal imbalance where the increase of sympathetic stimulation as a physiological response to physical exercise was potentiated by caffeine, as suggested by Yeragani *et al*. which attributes the highest values of LF power, during exhaustive exercise on a cycloergometer after caffeine consumption, to increase the responsiveness of sympathetic tone to heart rate^9^. No significant differences were found in the frequency domain indices when the values of the recovery moments with the resting value were compared, suggesting that these indices are not sensitive to identifying the changes observed using HRV indexes in the time domain.

Regarding global HRV, caffeine seems to have an influence, since the SDNN and SD2 indices had a time effect. The SDNN index presented higher values in the placebo protocol, reaching higher values during rest, mainly in the last moments (Rec4 to Rec6), which can be attributed to greater relaxation in the volunteers, who were in the supine position. This alteration was absent in the caffeine protocol, which may be related to caffeine’s stimulating capacity as mentioned above.

In our study, the SD2 index in the caffeine protocol presented the effect of time in relation to its basal value in Rec1. We attributed this to a possible increase in the responsiveness of the sympathetic component over the heart rate, as well as the delay of vagal re-entry after exercise, resulting in a higher sympathetic modulation and lower parasympathetic modulation, although such an effect was not observed in SDNN. This influence of caffeine on the response of the parasympathetic component of heart rate was studied by Jammes *et al*., who investigated HR responses after caffeine injection and found that caffeine was able to suppress the bradycardia induced by direct vagal stimulation in rats^28^. This would be attributed to the action of caffeine as an adenosine receptor blocker.

Because caffeine is a substance consumed to such a great extent^1^, there is a need for more studies to be carried out to identify its influences in different populations, establishing its benefits as well as the risks attributed to its use. Our study raises some important methodological points. Investigation sample was composed of healthy young men in order to avoid the influence of sexual hormones. For this reason, our results cannot be applied to female groups because of changes in caffeine metabolism during the menstrual cycle^29^ or to individuals with cardiac diseases who use drugs that affect the ANS. However, the selection of the sample, which was performed using rigorous exclusion criteria, strengthens our findings.

The results obtained can be used as a basis for future research about the impact of caffeine on post-exercise recovery in different populations, since it exposes influences in healthy young men during recovery following moderate aerobic exercise. We did not evaluate plasma catecholamine concentrations or the sympathetic nerve activity; however, we used HRV, a simple non-invasive method and one of the most promising quantitative markers of autonomic heart rate balance^17^. No respiratory control was carried out during the experiments, however, volunteers were instructed to breathe normally and remain awake during rest.

Regarding the dose of caffeine, although we did not take into consideration the volunteers’ body weight and habitual consumption of caffeine, the dose used was equal to the average amount consumed and within the recommended daily consumption. Further, they were instructed not to eat caffeine-containing foods for 24 hours before the protocols were performed^1^. Additionally, Corti *et al*. showed that acute responses are independent of habitual caffeine consumption, which could minimize this limitation^6^.

In conclusion, based on our findings, caffeine ingestion prior to moderate aerobic exercise delays recovery of the parasympathetic component of autonomic heart rate control, as well as the recovery of BP at baseline levels in young male participants.
